# Combined Branch Retinal Artery and Central Retinal Vein Occlusion: A Systematic Review

**DOI:** 10.3390/vision7030051

**Published:** 2023-07-28

**Authors:** Antonio Pinna, Angelo Zinellu, Rita Serra, Giacomo Boscia, Lorenza Ronchi, Stefano Dore

**Affiliations:** 1 Department of Medicine, Surgery and Pharmacy, University of Sassari, 07100 Sassari, Italy; stefanodore@hotmail.com; 2 Ophthalmology Unit, Azienda Ospedaliero-Universitaria di Sassari, 07100 Sassari, Italy; loreronchi@icloud.com; 3 Department of Biomedical Sciences, University of Sassari, 07100 Sassari, Italy; azinellu@uniss.it (A.Z.); rita.serra@ymail.com (R.S.); 4 Eye Clinic Section, Department of Surgical Sciences, University of Turin, 10126 Turin, Italy; bosciagiacomo@gmail.com

**Keywords:** branch retinal artery obstruction, central retinal vein occlusion, combined, management, systematic review

## Abstract

We performed a systematic review and analyzed the current available data on branch retinal artery occlusion (BRAO) with simultaneous central retinal vein obstruction (CRVO), a rarely described occurrence. MEDLINE/PubMed and ISI Web of Sciences searches were performed according to MOOSE guidelines. Studies were considered eligible if they (1) described patients with simultaneous BRAO + CRVO and (2) had been published in peer-reviewed journals. We initially identified 239 records from databases. Ultimately, only 19 reports met the selection criteria. Twenty-nine patients (15 men, 14 women; mean age 43 ± 15 years) were analyzed. Seventeen (59%) patients presented vascular risk factors. Mean visual acuity at onset and final visual outcome were 20/83 and 20/45, respectively, an insignificant improvement. Vision improved in 48% of cases. A marked heterogeneity in treatment approach was found. Eight (28%) patients received no therapy, whereas for 21 (72%) a large variety of topical and/or systemic drugs was given. In the treated group, mean visual acuity at onset and final visual outcome were 20/90 and 20/44, respectively, a not statistically significant improvement. Results suggest that combined BRAO + CRVO occurs at a younger age than isolated BRAO or CRVO. At present, there is insufficient evidence to support any specific management to improve vision in simultaneous BRAO + CRVO.

## 1. Introduction

Branch occlusion of the central retinal artery (BRAO) with simultaneous obstruction of the central retinal vein (CRVO) is a rarely described occurrence [1,2,3,4,5,6,7,8,9,10,11,12,13,14,15,16,17,18,19], usually presenting with sudden, painless central and/or altitudinal vision loss. This condition, characterized by retinal whitening in the territory of the affected retinal arterial branch together with typical CRVO features (i.e., dilated, tortuous retinal veins, diffuse retinal hemorrhages, cotton wool spots, macular and disc edema, generalized delay in arteriovenous transit on fluorescein angiography), differs clinically from CRVO associated with the simultaneous occlusion of either the central retinal artery or a cilioretinal artery [20,21,22].

Although BRAO and CRVO share may underlying systemic risk factors, such as systemic arterial hypertension, diabetes mellitus, atherosclerotic cardiovascular disease, and coagulopathies [1,20,21,22], the pathogenesis of combined BRAO + CRVO is still to be determined. It has been postulated either that CRVO might be the initiating event followed by the external compression of an adjacent branch retinal artery by the ensuing optic disc swelling or that BRAO might be the inciting event followed by a low-flow state in the central retinal vein [1].

Very few cases of combined BRAO + CRVO have been reported in the last three decades [1,2,3,4,5,6,7,8,9,10,11,12,13,14,15,16,17,18,19], even though CRVO is a common vascular disorder.

Little is known about the demographics, visual acuity at onset, final visual outcome, vascular risk factors, and management of combined BRAO + CRVO. The purpose of this systematic review was to analyze the current available data on this uncommon retinal vascular disorder.

## 2. Materials and Methods

### 2.1. Eligibility Criteria for Considering Studies for This Review

Studies were considered eligible for this systematic review if they met the following criteria: (1) they described patients with simultaneous BRAO and CRVO, (2) they had been published as articles or letters in peer-reviewed journals.

### 2.2. Search Methods for Identifying Studies

A literature review was performed according to Meta-analysis of Observational Studies in Epidemiology (MOOSE) guidelines for Meta-Analyses and Systematic Reviews of Observational Studies. Eligible studies were identified by searching MEDLINE/PubMed using this strategy: (explode ‘branch central artery occlusion’ [All Fields] AND ‘central retinal vein occlusion’ [All Fields] AND ‘combined’ [All Fields]) and (explode ‘branch central artery occlusion’ [All Fields] AND ‘central retinal vein occlusion’ [All Fields] AND ‘simultaneous’ [All Fields]). A similar strategy was used in searches on ISI Web of Science: search (branch retinal artery occlusion) AND (central retinal vein occlusion) AND (combined OR simultaneous). On each database, the search was limited to studies on humans published up to and including June 2023. 

### 2.3. Study Selection

Abstracts were screened independently by four investigators (A.P., R.S., L.R., S.D.) to establish whether studies were likely to provide relevant data based on the above-mentioned eligibility criteria. If the abstracts were considered to be relevant, full articles were obtained and examined. Any disagreement between the reviewers in the abstract review or following article selection for full-text review was resolved by discussion. Furthermore, the reference lists of all relevant articles were screened for additional articles. 

### 2.4. Data Collection and Risk of Bias Assessment

Eligible studies were assessed independently by three reviewers (A.P., R.S., A.Z.) using a structured form to extract information about the study (country and year of publication) and study subjects (number of cases, age, eye and retinal arterial branch involved, visual acuity at presentation, final visual outcome with time after symptom onset, vascular risk factors, management).

### 2.5. Data Synthesis and Analysis

Student’s *t*-test, Wilcoxon test, and Fisher’s exact test were used, as appropriate. Bonferroni correction for multiple comparisons was used to reduce type 1 error rate. *p* < 0.05 was considered to be statistically significant. Statistical analysis was performed with commercial software (STATA Statistical Software: Release 14 for Windows, StataCorp. 2015, College Station, TX, USA).

## 3. Results

**Figure 1:**
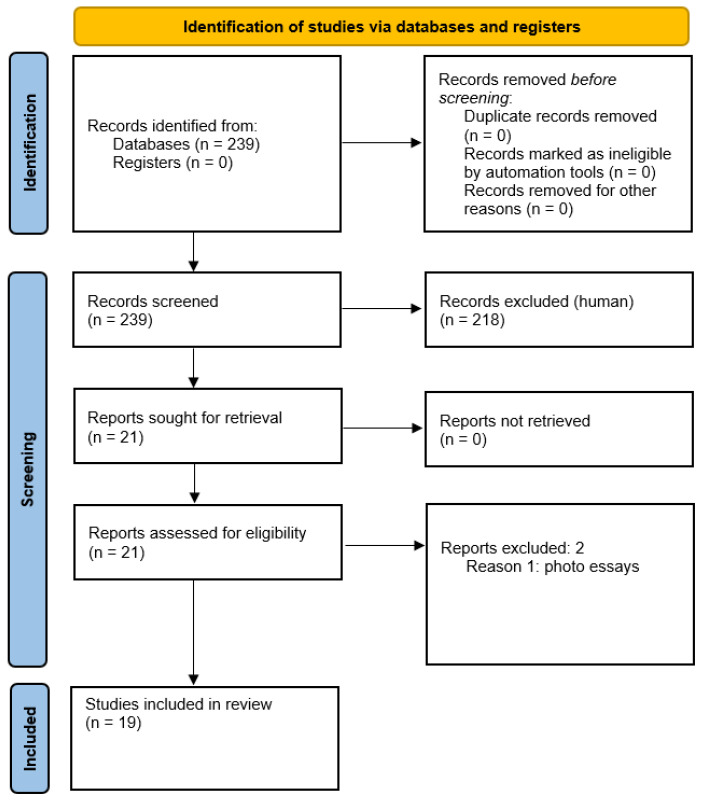
PRISMA 2020 flow diagram outlining the selection process for the inclusion of the studies in the systematic review [23].

The Preferred Reporting Items for Systematic Reviews and Meta-Analyses (PRISMA) flow diagram of the selection process is shown in Figure 1 [23]. 

We initially identified 239 records from databases. Of these, 218 were excluded after the first screening, because they were irrelevant for our systematic review. Twenty-one reports on combined BRAO and CRVO were sought for retrieval. 

Twenty-one reports were assessed for eligibility; two of them were excluded, because they were photo essays with insufficient demographic and clinical information [24,25]. In total, 19 of the 21 initially identified studies, published between 1990 and 2022, met the selection criteria and were finally included in the review. Demographics, visual acuity at onset, final visual outcome with time post-symptom onset, known risk vascular factors, and therapy in patients with combined BRAO + CRVO are shown in Table 1.

Generally, a diagnosis of BRAO was based on the ophthalmoscopic detection of an area of intense retinal whitening along the course of the affected arterial branch. Fluorescein angiography showed evidence of absence or marked stasis of circulation in the involved arteriole. 

A diagnosis of CRVO was based on the fundoscopic detection of typical features, including dilated, tortuous retinal veins, diffuse retinal hemorrhages, cotton wool spots, and macular and disc edema. Fluorescein angiography disclosed a generalized delay in arteriovenous transit.

We found that Spectral Domain Optical Coherence Tomography (SD-OCT) was performed only in the more recent reports, dating from 2014 onward [10,11,12,13,14,15,16,18,19]. SD-OCT revealed hyper-reflective band-like lesions at the level of the inner nuclear layer in the areas of macular whitening, a finding known as paracentral acute middle maculopathy (PAMM).

Five studies (11 patients) were from the Americas [1,4,7,13,17], nine (13 patients) from Asia [5,8,10,11,12,14,16,18,19] and five (5 patients) from Europe [2,3,6,9,15].

A total of 29 patients (mean age 43 ± 15 years) were analyzed. There were 15 men (mean age 46 ± 17 years) and 14 women (mean age 40 ± 13 years). No statistically significant differences were found in terms of mean age between patients living in different geographical areas.

Combined BRAO + CRVO affected the right eye in 19 (66%) patients and the left in 10 (34%); no statistically significant differences were found in terms of gender distribution.

The involvement of the arterial branches was documented only in the temporal retina (i.e., 15 supero- and 14 infero-temporal BRAOs); no gender differences were observed.

All the BRAOs originated at the level of the optic disc and affected arterioles supplying the macula. No intra-arterial emboli were visualized. Apart from the cases described by Bajare et al. [7] and Kumar et al. [18], all the other reports showed no evidence of retinal vasculitis [1,2,3,4,5,6,8,9,10,11,12,13,14,15,16,17,19].

Fluorescein angiography disclosed non-ischemic CRVO in 21 (72%) patients; the remaining eight (28%) had ischemic CRVO.

Mean visual acuity at onset was 0.24 ± 0.3 decimal equivalent (D.E.; 20/83 Snellen equivalent), whereas final visual outcome was 0.45 ± 0.4 D.E. (20/45 Snellen equivalent), an insignificant improvement (Bonferroni-corrected *p* = 0.49).

Overall, some degree of visual improvement was documented in 48% of patients. In 24% of eyes vision remained unchanged, and in 14% vision worsened.

Vascular risk factors, including systemic hypertension [1,14], diabetes mellitus [1], inherited plasminogen deficiency and high lipoprotein(a) levels [2], hyperhomocysteinemia [5,11,12,17], systemic lupus erythematosus (SLE) [18], SARS-CoV-2 infection associated with B cell acute lymphoblastic leukemia in remission [19], chronic hepatitis C under treatment with interferon [4,6,7,8], and multiple sclerosis under treatment with interferon [9], were reported in 17 patients. The remaining 12 had no underlying vascular risk factors; however, in two of them, combined BRAO + CRVO occurred after intense physical activity, such as skiing and running a half marathon [1,2,3,4,5,6,7,8,9,10,11,12,13]. Men and women were similarly distributed between the two groups.

Mean final visual acuity at onset was 0.45 ± 0.36 D.E. (20/45 Snellen equivalent) in the group without underlying vascular risk factors and 0.14 ± 0.2 D.E. (20/145 Snellen Equivalent) in the group with vascular risk factors, a not statistically significant difference (Bonferroni-corrected *p* = 0.096).

Mean final visual outcome was 0.65 ± 0.38 D.E. (20/32 Snellen equivalent) in the group without vascular risk factors and 0.33 ± 0.34 D.E. (20/60 Snellen Equivalent) in the group with vascular risk factors. In both groups, there was visual improvement; however, this change in visual acuity from symptom onset to final measurement was not statistically significant.

A marked heterogeneity in the treatment approach was found. Overall, eight (28%) patients received no therapy, whereas in the remaining 21 (72%) a large variety of topical and/or systemic drugs was given.

In the group who received some treatment, mean visual acuity at presentation and final visual outcome were 0.21 ± 0.3 D.E. (20/90 Snellen equivalent) and 0.44 ± 0.4 D.E. (20/45 Snellen equivalent), respectively, a not statistically significant improvement (Bonferroni-corrected *p* = 0.42).

Similarly, in the group not treated, visual improvement from 0.36 ± 0.3 D.E. (20/55 Snellen equivalent) to 0.42 ± 0.4 D.E. (20/48 Snellen equivalent) was not statistically significant. No differences were found between the two groups in terms of initial visual acuity and final visual outcome.

## 4. Discussion

Simultaneous vascular occlusion affecting the retinal vein and artery is an unusual occurrence. Traditionally, retinal vein obstructions are divided into CRVO, branch retinal vein occlusion (BRVO), and hemi-central retinal vein occlusion (HCRVO). Retinal artery occlusions are classified into central retinal artery occlusion (CRAO), BRAO, and cilioretinal artery occlusion (CLRAO). The simultaneous vein and artery obstruction could be any permutation and combination, such as CRVO + CRAO, CRVO + BRAO, BRVO + CRAO, BRVO + BRAO, and CLRAO + CRVO. Most patients suffer from CRVO + CRAO, and occasionally with CLRAO or BRAO [14,21,22]. The combination BRVO + BRAO is less common.

BRAO with simultaneous CRVO, the topic of the present review, is a rarely documented condition [1,2,3,4,5,6,7,8,9,10,11,12,13,14,15,16,17,18,19]. In a recent study from India [14], combined BRAO + CRVO accounted for only 0.02% (1/5151) of all cases of retinal vascular occlusions seen at a tertiary eye care center during a three-year period.

A variety of pathological mechanisms have been implicated for these combined occlusions. A sudden increase in intraluminal retinal capillary bed pressure secondary to CRVO could lead to CLRAO [22]. Likewise, a decrease in the perfusion pressure of the cilioretinal and retinal arteries may lead, in turn, to decreased retinal circulation and subsequent venous stasis and thrombosis. Many systemic co-morbidities associated with retinal vein occlusions (e.g., systemic arterial hypertension, atherosclerosis, hyperlipidemia, diabetes mellitus, coagulopathies, hyperviscosity blood disorders, systemic vasculitis) could damage the adjacent artery to cause combined vascular occlusion.

Combined BRAO + CRVO is a distinct clinical entity from CRVO associated with CLRAO [21,22]. In eyes with a cilioretinal artery, it can be difficult to differentiate the two retinal vascular disorders, unless very high-quality fluorescein angiography with early frame can be evaluated. In patients with combined CLRAO + CRVO examined shortly after the onset, a typical oscillating blood column in the cilioretinal artery can be seen in fluorescein angiograms. While the precise mechanism of simultaneous BRAO + CRVO is still to be established, the pathogenesis of combined CLRAO + CRVO is believed to be caused by the transient hemodynamic blockage of the cilioretinal artery due to a sudden sharp rise in intraluminal pressure in the retinal capillary bed above the level of that in the cilioretinal artery [22].

We performed this systematic review to analyze the current available data on combined BRAO + CRVO. The review criteria identified 19 reports, for a total of 29 patients with data included in the results. We obtained results for 12 hypothesis tests, and the *p* value threshold for multiple comparisons was adjusted by using Bonferroni’s correction.

The mean age at presentation for combined BRAO + CRVO was 43 years, a much younger age than that reported for isolated BRAO (early 60s) [26,27] and isolated CRVO (6–7th decade) [28], but a similar age to that found in patients with CLRAO + nonischemic CRVO [22]. Patients living in different geographical areas showed a similar mean age at onset. A gender predilection does not seem to exist, and right eyes appear to be affected more commonly. The obstruction of the arterial branches was documented exclusively in the temporal retina. This finding might be due to an observer bias, where the examiner looks more closely at the macula, compared to the nasal retina. Furthermore, patients will be more symptomatic of a temporal BRAO.

The vast majority of cases had non-ischemic CRVO. The non-ischemic nature of the CRVO could be an important factor in patients with concomitant BRAO + CRVO, yielding a relatively good prognosis.

PAMM is an OCT finding observed in patients with retinal capillary ischemia and unspecific persistent scotomas, characteristically appearing as a placoid, hyperreflective band at level of the inner nuclear layer. It can occur as an isolated phenomenon or a complication of an underlying retinal vasculopathy, such as central artery or vein occlusions [27,28,29]. Regardless of the vessel involved, PAMM is an early biomarker of the ischemic cascade in general. Our systematic review disclosed that PAMM can develop early in eyes with combined BRAO + CRVO [10,11,12,13,14,15,16,18,19].

In patients with simultaneous BRAO + CRVO, mean visual acuity at onset was 20/83, whereas final visual outcome was 20/45, a not significant improvement after Bonferroni’s correction. These results are different from those reported by Hayreh et al. [19] for CLRAO + nonischemic CRVO, which showed a severe visual loss at onset (20/400 or less) in only 20% of cases and a good visual outcome (20/30 or better) in 90%. However, it is important to clarify that in the study by Hayreh et al. [22] only 5 out of 30 non-ischemic CRVO patients had cilioretinal infarction involving some, or all, of the fovea. This can explain why vision was generally good and improved.

Vascular risk factors were found in 59% of the patients with combined BRAO + CRVO. These included systemic arterial hypertension [1,14], diabetes mellitus [1], inherited plasminogen deficiency and high lipoprotein(a) levels [2], elevated serum levels of homocysteine [5,11,12,17], SLE [18], SARS-CoV-2 infection associated with B cell acute lymphoblastic leukemia in remission [19], chronic hepatitis C under treatment with interferon [4,6,7,8], and multiple sclerosis under treatment with interferon [9].

Notably, five (17%) out of 29 patients with combined BRAO + CRVO were receiving interferon therapy. This finding seems to be interesting, because interferon-induced retinopathy is a well-known complication of interferon therapy [30]. Usually, this condition is characterized by flame-shaped hemorrhages and cotton wool spots, but more severe side effects, including non-arteritic anterior ischemic optic neuropathy and mixed retinal vascular occlusion, have been described [30].

Chronic occlusive changes in small retinal arterioles are responsible for lupus-associated retinopathy, which consists of cotton-wool spots, perivascular hard exudates, retinal hemorrhages, and optic disc edema. The prevalence of lupus retinopathy ranges from 3% in well-controlled subjects to 29% in patients with severe active SLE [18]. Recently, Kumar et al. [18] have reported two cases of atypical presentations of lupus retinopathy, characterized by simultaneous BRAO + CRVO (ischemic and non-ischemic type).

In the report by Panigrahi et al. [19], the prothrombotic state associated with SARS-CoV-2 infection had most probably produced a thrombosis within both the arterial and the venous circulation, resulting in combined CRVO + BRAO.

Like BRAO, acute, symptomatic simultaneous BRAO + CRVO represents an urgent ophthalmic condition requiring prompt systemic evaluation. Indeed, BRAO + CRVO may be an important clinical indicator of an embolic, inflammatory, or other process requiring a systemic medical evaluation that is both urgent and targeted to the patient’s presentation and medical history. Overall, in younger patients (under the age of 50 years), a detailed workup for hypercoagulability or vasculitis is mandatory [27]. In older patients (aged 50 years or older), an embolic workup is warranted [28].

We found that both groups with and without vascular risk factors showed visual improvement; however, this change in visual acuity from symptom onset to final measurement did not meet statistical significance.

There has been only one major series of combined BRAO + CRVO, reported in 1990 by Duker et al. [1], who described seven patients with this unusual condition. These authors classified combined BRAO + CRVO into two groups: a group of three patients without underlying systemic risk factors and a second group of four patients with vasculopathic risk factors. In the first group, visual outcome was generally good (20/30 or better); no therapy was instituted in two cases, whereas one patient was treated with intravenous methylprednisolone. In the second group, two patients had a significant improvement in visual acuity (20/20 and 20/40, respectively) after treatment with oral acetylsalicylic acid alone or in combination with a carbonic anhydrase inhibitor. Conversely, two other patients, one treated with oral acetylsalicylic acid and dipyridimole, the other left with no treatment, had a poor visual outcome (counting fingers). These authors concluded that there was no evidence that therapy altered the natural course of the disease. However, this statement was made before the advent of the new intravitreal drugs for the treatment of CRVO-related macular edema.

In our systematic review, we found a marked heterogeneity in the treatment approach, basically due to the lack of official guidelines. A large variety of topical and/or systemic drugs was given to 72% of the patients with combined BRAO + CRVO. Our analysis demonstrates that the group who received some treatment showed a much greater visual improvement (from 20/90 to 20/45) than the group not treated (from 20/55 to 20/48); however, after Bonferroni’s correction this change was not statistically significant.

Dexamethasone intravitreal implant (Ozurdex^®^) has been shown to be beneficial in the treatment of any subtype of CRVO [31]. Recently, Ozturk et al. [11] and Arrigo et al. [15] reported a good visual recovery in two patients with simultaneous BRAO + CRVO and macular edema who received a dexamethasone implant. Similarly, in another patient, visual improvement was obtained after a periocular injection of triamcinolone acetonide [10]. Overall, these data suggest that the prompt treatment of BRAO + CRVO-associated macular edema is very important for visual prognosis. It has been postulated that intravitreal steroids may reduce macular edema, thus contributing to venous engorgement reduction and, consequently, arterial perfusion improvement [15].

In the setting of elevated plasma homocysteine, oxidative stress and the activation of proinflammatory factors may play a role in the pathogenesis of atherosclerosis. Furthermore, homocysteine may act as a weak prothrombotic factor, thus predisposing patients to retinal vascular occlusions. Oral folic acid supplementation has been found to be effective in young patients with combined BRAO + CRVO and hyperhomocysteinemia, resulting in the normalization of plasma homocysteine values and vision improvement [5,12].

In patients with simultaneous BRAO + CRVO under treatment with interferon for hepatitis C [4,6,7,8] or multiple sclerosis [9], the discontinuation of interferon therapy failed to yield any visual improvement.

Bevacizumab or ranimizumab were used in four patients (three with ischemic CRVO, one with non-ischemic CRVO) [7,14,18]. In these cases, the anti-VEGF therapy was ineffective in improving vision.

Overall, from the available data, there is insufficient evidence to support any specific treatment to improve vision in combined BRAO + CRVO. Intravitreal dexamethasone implant may be beneficial in selected cases, presenting significant CRVO-related macular edema.

The most important limitation of our systematic review is due to the small number of studies (all case series or reports) on this topic available in the literature. Consequently, with a study sample of only 29 patients, it was not possible to perform a meta-analysis. Our investigation reports results for several hypothesis tests with *p* values adjusted by Bonferroni’s correction for multiple comparisons; however, because of the paucity of data, conclusions cannot be generalized. Nevertheless, to the best of our knowledge, our systematic review is the largest study on combined BRAO + CRVO.

## 5. Conclusions

BRAO + CRVO constitutes a rare clinical entity, usually presenting with sudden, painless central and/or altitudinal vision loss. Ophthalmoscopic examination supplemented with fluorescein angiography and OCT are essential to establish the correct diagnosis. BRAO + CRVO seems to occur at a younger age than isolated BRAO or CRVO. A gender predilection does not seem to exist. Occlusion of the arterial branches was documented exclusively in the temporal retina. Approximately 60% of the patients with simultaneous BRAO + CRVO had vascular risk factors. Some degree of visual improvement was found in half of cases. At present, there is insufficient evidence to support any specific management to improve vision in simultaneous BRAO + CRVO. Like BRAO, acute, symptomatic combined BRAO + CRVO represents an urgent ophthalmic condition requiring prompt systemic medical evaluation to exclude microembolism, hypercoagulability, or vasculitis. Further future studies are warranted to improve our knowledge of this uncommon retinal vascular disorder.

## Figures and Tables

**Table 1:** Demographics, visual acuity at onset, final visual outcome, known risk vascular factors, and therapy in patients with combined BRAO + CRVO. M = male; F = female; R.E. = Right Eye; L.E. = Left Eye; C.F. = Counting fingers; H.M. = Hand Movements; D.E. = Decimal equivalent; () * = time after symptom onset; LMWH = low molecular weight heparin.

Patient	Gender/Age (Years)	Eye & Arterial Branch	CRVO	Visual Acuity at Onset	Final Visual Outcome	Vascular Risk Factors	Therapy
1 (Duker et al. 1990, US) [1]	F/24	L.E.; infero-temporal	Nonischemic	20/2000 (C.F.) [0.01 D.E.]	20/30 [0.66 D.E.] (7 months) *	No	Systemic steroids
2 (Duker et al. 1990, US) [1]	M/42	R.E.; supero-temporal	Nonischemic	20/70 [0.29 D.E.]	20/25 [0.8 D.E.] (2 months) *	No	No therapy
3 (Duker et al. 1990, US) [1]	M/20	R.E.; supero-temporal	Nonischemic	20/20 [1 D.E.]	20/20 [1 D.E.] (3 months) *	No	No therapy
4 (Duker et al. 1990, US) [1]	M/62	L.E.; supero-temporal	Nonischemic	20/2000 (C.F.) [0.01 D.E.]	20/40 [0.5 D.E.] (6 years) *	Yes	Aspirin and carbonic anydrase inhibitor
5 (Duker et al. 1990, US) [1]	M/65	L.E.; infero-temporal	Nonischemic	20/20 [1 D.E.]	20/20 [1 D.E.] (not reported) *	No	Aspirin
6 (Duker et al. 1990, US) [1]	F/53	R.E.; infero-temporal	Ischemic	20/20,000 (H.M.) [0.001 D.E.]	20/2000 (C.F.) [0.01 D.E.] (4 years) *	Yes	Aspirin and dipyridimole
7 (Duker et al. 1990, US) [1]	M/85	R.E.; supero-temporal	Ischemic	20/2000 (C.F.) [0.01 D.E.]	20/2000 (C.F.) [0.01 D.E.] (2 years) *	No	No therapy
8 (Tavola et al. 1995, Italy) [2]	F/45	R.E.; supero-temporal	Ischemic	20/2000 (C.F.) [0.01 D.E.]	20/2000 (C.F.) [0.01 D.E.] (3 months) *	Yes	Warfarin
9 (Singh 2001, UK) [3]	F/28	R.E.; supero-temporal	Nonischemic	20/60 [0.33 D.E.]	20/125 [0.16 D.E.] (9 months) *	No	No therapy
10 (Rubio & Charles 2003, US) [4]	M/51	L.E.; infero-temporal	Nonischemic	20/2000 (C.F.) [0.01 D.E.]	20/20000 (H.M.) [0.001 D.E.] (not reported) *	Yes	Aspirin and topical timolol
11 (Özdek et al. 2004, Turkey) [5]	M/38	R.E.; infero-temporal	Nonischemic	20/60 [0.33 D.E.]	20/20 [1 D.E.] (6 months) *	Yes	Mannitol infusion, acetazolamide, folic acid
12 (Özdek et al. 2004, Turkey) [5]	M/25	R.E.; infero-temporal	Nonischemic	20/50 [0.4 D.E.]	20/25 [0.8 D.E.] (5 months) *	Yes	Mannitol infusion, acetazolamide, folic acid
13 (Nicolò et al. 2005, Itay) [6]	M/29	L.E.; infero-temporal	Ischemic	20/320 [0.06 D.E.]	20/320 [0.06 D.E.] (not reported) *	Yes	No therapy
14 (Rosenbaum et al. 2010, US) [17]	F/14	L.E.; infero-temporal	Nonischemic	20/2000 (C.F.) [0.01 D.E.]	20/50 [0.4 D.E.] (4 weeks) *	Yes	Vitamin B6 supplementation
15 (Bajare et al. 2011, Colombia) [7]	F/45	L.E.; supero-temporal	Ischemic	20/2000 (C.F.) [0.01 D.E.]	20/20000 (H.M.) [0.001 D.E.] (3 years) *	Yes	Intravitreal bevacizumab
16 (Watanabe et al. 2012, Japan) [8]	M/62	R.E.; supero-temporal	Nonischemic	20/400 [0.05 D.E.]	20/400 [0.05 D.E.] (16 months) *	Yes	Eye-drops for IOP reduction
17 (Jenisch et al. 2012, Germany) [9]	F/44	R.E.; infero-temporal	Nonischemic	20/500 [0.04 D.E.]	20/25 [0.8 D.E.] (6 weeks) *	Yes	Aspirin
18 (Karapetyan et al. 2014, China) [10]	F/51	R.E.; supero-temporal	Nonischemic	20/320 [0.06 D.E.]	20/60 [0.33 D.E.] (4 weeks) *	No	Periocular triamcinolone acetonide
19 (Ozturk et al. 2015, Turkey) [11]	M/30	R.E.; supero-temporal	Nonischemic	20/20,000 (H.M.) [0.001 D.E.]	20/25 [0.8 D.E.] (6 months) *	Yes	Mannitol infusion, ant. chamber paracentesis, Ozurdex^®^
20 (Parchand 2016, India) [12]	F/30	R.E.; supero-temporal	Nonischemic	20/80 [0.25 D.E.]	20/40 [0.5 D.E.] (6 months) *	Yes	Folic acid, pyridoxine
21 (Coca et al. 2017, US) [13]	M/47	R.E.; infero-temporal	Nonischemic	20/20 [1 D.E.]	20/20 [1 D.E.] (1 month) *	No	Topical timolol
22 (Raval et al. 2020, India) [14]	M/52	L.E.; infero-temporal	Ischemic	20/2000 (C.F.) [0.01 D.E.]	20/20000 (H.M.) [0.001 D.E.] (9 months) *	Yes	Intravitreal bevacizumab, panretinal photocoagulation
23 (Arrigo et al. 2021, Italy) [15]	M/51	L.E.; infero-temporal	Ischemic	20/40 [0.5 D.E.]	20/20 [1 D.E.] (2 years) *	No	Ozurdex^®^
24 (Goel 2021, India) [16]	F/52	R.E.; supero-temporal	Nonischemic	20/60 [0.33 D.E.]	20/40 [0.5 D.E.] (6 weeks) *	No	No therapy
25 (Goel 2021, India) [16]	F/48	R.E.; supero-temporal	Nonischemic	20/40 [0.5 D.E.]	Not reported	No	No therapy
26 (Goel 2021, India) [16]	F/45	R.E.; supero-temporal	Nonischemic	20/60 [0.33 D.E.]	Not reported	No	No therapy
27 (Kumar et al. 2021, India) [18]	F/51	R.E.; infero-temporal	Ischemic	20/40 [0.5 D.E.]	20/60 [0.33 D.E.] (6 months) *	Yes	Systemic steroids, LMWH, intravitreal ranimizumab
28 (Kumar et al. 2021, India) [18]	M/34	L.E.; infero-temporal	Nonischemic	20/20,000 (H.M.) [0.001 D.E.]	20/20000 (H.M.) [0.001 D.E.] (6 months) *	Yes	Systemic steroids, LMWH, intravitreal ranimizumab
29 (Panigrahi et al. 2022, India) [19]	F/23	R.E.; superotemporal	Nonischemic	20/30 [0.66 D.E.]	Not reported	Yes	Systemic anticoagulants

## Data Availability

Data available on request.
